# Alzheimer's disease biomarkers and memory performance in a Brazilian population

**DOI:** 10.1002/alz70857_106842

**Published:** 2025-12-26

**Authors:** Elisa de Paula, França Resende, Leonardo Cruz de Souza, Paulo Caramelli, Karen Luíza Meira, Leandro Boson Gambogi, Clarisse Vasconcelos Friedlaender, Luciano Inácio Mariano, Viviane Amaral‐Carvalho, Etelvina Lucas dos Santos, Larissa de Souza, Larissa Salvador, Patricia Henrique, Karoline Carvalho Carmona, Mauro Cunningham, Rogerio Gomes Beato, Jessica Tossati, Jessica Diniz Pereira, Karina Braga Gomes, Claudia Natalia Ferreira

**Affiliations:** ^1^ Universidade Federal de Minas Gerais, Belo Horizonte, Minas Gerais, Brazil; ^2^ Federal University of Minas Gerais, Belo Horizonte, Minas Gerais, Brazil; ^3^ Faculty of Medicine ‐ Federal University of Minas Gerais, Belo Horizonte, Minas Gerais, Brazil; ^4^ Universidade Federal da Bahia, Belo Horizonte, Belo Horizonte, Brazil; ^5^ Federal University of Minas Gerais, Belo Horizonte, Brazil; ^6^ Universidade Federal de Minas Gerais, Belo Horizonte, Brazil; ^7^ Universidade Federal de Minas Gerais, Belo Horizonte, Minas ∖gerais, Brazil; ^8^ Universidade Federal da Bahia, Belo Horizonte, Minas Gerais, Brazil

## Abstract

**Background:**

Understanding the relationship between Alzheimer's disease (AD) biomarkers and cognitive performance in diverse populations is essential to uncover how social and demographic factors influence brain pathology and clinical manifestations. Studying these associations in underrepresented groups, can enhance the global applicability of diagnosis using AD biomarkers and therapeutic strategies.

**Method:**

This study included 72 participants with an average of 8.9 [SD ±5.1] years of education, ranging from 0 to 21 years, from a tertiary memory clinic in Brazil. All of them underwent CSF biomarker analysis and a neuropsychological evaluation as part of their diagnostic workup. Among the participants, 30 were diagnosed with AD (39 men and 33 women; mean age 65.1 years [SD ±8.2], range 43–83) with CSF biomarker levels of *p*‐tau 87.0 (±41.7), total tau 765 (±611), and AB‐42 579 (±195); 16 had behavioral variant frontotemporal dementia (bvFTD) (13 men and 3 women; mean age 64.3 years [SD ±7.9], range 43–76) with *p*‐tau 49.1 (±21.2), total tau 405 (±346), and AB‐42 875 (±334); seven had mild cognitive impairment (MCI) (1 men and 6 women; mean age 62.3 years [SD ±9.3], range 50–78) with *p*‐tau 63.4 (±36.7), total tau 315 (±322), and AB‐42 1050 (±330); and 19 had dementia without a specific classification (11 men and 8 women; mean age 63.8 years [SD ±9.7], range 43–82) with *p*‐tau 51.8 (±24.7), total tau 389 (±262), and AB‐42 941 (±379).

**Result:**

Our analysis revealed significant correlations between AD CSF biomarkers and cognitive performance. Total tau was negatively correlated with verbal memory performance (z_scoreRAVLT, Spearman's rho = ‐0.265, *p* =  0.025), while beta‐amyloid 42 (AB‐42) was positively correlated with verbal memory (z_scoreRAVLT, Spearman's rho = 0.266, *p* =  0.024). However, AB‐42 did not show significant correlation with global cognitive function (z_scoreMattis, Spearman's rho = 0.210, *p* =  0.076). No significant correlations between phosphorylated tau and cognitive measures emerged, as well.

**Conclusion:**

In a Brazilian population with a diverse educational level, total tau biomarkers showed significant correlations with verbal memory tests. However, phosphorylated tau and AB‐42 did not demonstrate such correlations. These findings suggest that the cognitive impairment does not follow amyloidosis, but seems to reflect neurodegeneration.

